# School-Based Interventions in Low Socioeconomic Settings to Reduce Obesity Outcomes among Preschoolers: A Scoping Review

**DOI:** 10.3390/nu11071518

**Published:** 2019-07-04

**Authors:** Megan Luybli, Hanna Schmillen, Mercedes Sotos-Prieto

**Affiliations:** 1 Division of Food Sciences and Nutrition, School of Applied Health Sciences and Wellness, Ohio University, Athens, OH 45701, USA; 2 The Diabetes Institute, Ohio University, Athens, OH 45701, USA; 3 Ohio University Libraries, Ohio University, Athens, OH 45701, USA; 4 Department of Environmental Health, Harvard T.H. Chan School of Public Health, 677 Huntington Avenue, Boston, MA 02115, USA; 5 Department of Preventive Medicine and Public Health, School of Medicine, Universidad Autónoma de Madrid, C/Arzobispo Morcillo, sn, 28029 Madrid, Spain

**Keywords:** Socioeconomic status, rural, school-based interventions, preschool, body mass index, waist circumference, skinfold, percent body fat, pediatric obesity, children

## Abstract

Pediatric obesity continues to grow globally, specifically in low-socioeconomic rural areas. Strategies that combat pediatric obesity have not yet been fully determined. While the implementation of some interventions in preschool (ages 2–5) populations have demonstrated successful results, others have proven to be inconclusive and less have focused specifically on low socioeconomic populations. This scoping review aims to examine the literature to study the effectiveness of the school-based interventions in low socioeconomic settings on adiposity-related outcomes among preschoolers. PubMed/MEDLINE and EBSCO (ERIC (Education Resource Information Center) and Food Science Source) were used to conduct the search strategy. A total of 15 studies were assessed that met the inclusion criteria: Studies that included school-based interventions; reported adiposity-related data; targeting preschoolers (2 to 5 years old) in rural/low socioeconomic/underserved/areas. Interventions were then described as successful or inconclusive based on the primary outcome. Nine out of the fifteen studies were labeled as successful, which had a reduction in adiposity-related outcomes (BMI (body mass index), BMI z-score, waist circumference, skinfold, percent body fat). Current evidence, although scarce, suggest that obesity outcomes can be targeted in low socioeconomic settings through school interventions with a multicomponent approach (nutrition and physical activity) and the inclusion of parents. Further research is needed to determine effective interventions, their efficacy, and their long-term outcomes.

## 1. Introduction

Pediatric obesity is a growing global health concern. According to data from World Health Organization (WHO) in 2016, 41 million children under 5 years old were overweight or obese [1]. Overweight and obesity are defined by the WHO as “abnormal or excessive fat accumulation that presents a risk to health.” Childhood obesity has been defined using different methods for instance, WHO Child Growth Standards [1], the Center for Disease Control (CDC) (that defines overweight as a body mass index (BMI) at or above the 85th percentile while obesity is a BMI at or above the 95th percentile) [2], and the International Obesity Task Force (IOTF) [3]. Despite previous reports showing a stabilization in obesity prevalence in the United States [4], an updated review using National Health and Nutrition Examination Survey (NHANES) data by Skinner et al. (2018) [5] demonstrated that there has been a significant increase in childhood obesity and severe obesity in children 2 to 5 years old since 2013–2014 NHANES report. And according to Skinner et al. (2008) [5], this trend is continuing to grow. Although obesity is affecting children all over the world, low- and middle-income areas are the most prone [1]. Children in households that live below the poverty level in the United States show obesity rates 2.7 times higher than the average [6]. Therefore, children in underserved areas are 20% to 60% more likely to be overweight or obese compared to those with high socioeconomic status (SES) [6]. This preventable, chronic disease has started to affect preschool-aged children. Increasing obesity rates indicate increased likelihood of developing comorbidities, such as type 2 diabetes and heart disease [7]. Data from the NHANES show the rate of obesity is 13.9% in preschool-aged children, ages 2 to 5 years old [8]. In comparison to the 1976–1980 NHANES survey, the prevalence of obesity has tripled [8]. Of all ages, the races that are most affected by obesity include American Indian/Alaskan Natives (18.0%), Whites (12.2%), Asian/Pacific Islanders (11.1%), African Americans (11.9%), and Latinos (17.3%) [2]. SES differences in BMI tend to emerge by four years old and increase with age [9].

Preschool may be one of the first settings where children learn behavioral norms with eating and developing eating habits that carry into adulthood [10]. Typically, there is a high preschool attendance rate in industrialized countries and therefore, school-based interventions (interventions delivered at children in an educational setting) may be most effective for the rural communities [11]. However, low SES represents an additional challenge to overcome obesity as evidence suggests that disadvantaged populations (such as low SES or rural communities) lack access to healthy foods and the resources to engage in physical activity (PA) (such as PA facilities or free activities) [7,12]. This increases the risk of poorer health choices, diet quality, and sedentary behaviors, which are all risk factors for obesity [8]. Thus, successful school interventions in such a setting may have a greater impact and may benefit at-risk populations, especially in the preschool ages.

Different school intervention studies that have been described in the literature make it apparent that implementing programs at such a young age may be the best way to tackle such a problem [8,13,14]. Although school-based intervention studies are increasing and presenting promising results, there seems to be a wide variety between the studies including the length of the study, the focus of the intervention (diet, PA, multicomponent), the target population of the intervention (children, environment, parents), and the school age or the socioeconomic level [15]. A recent review of 25 studies, based on interventions in primary school age, found that most of the studies evaluated had positive results on BMI, PA, and sedentary behavior but not with regard to nutritional behavior [16]. Multiple studies highlighted the parental involvement in the intervention to be a key piece in successful interventions [13,17,18,19,20,21,22,23,24]. Likewise, studies that include a multicomponent intervention seem to have positive results for BMI reduction, another consideration is the effect size of BMI reduction which evidence shows is still limited. However, other studies showed inconclusive results on BMI [12,25,26,27,28,29]. For example, an intervention in Germany that lasted for six months consisted of nutrition education sessions that targeted fruit and vegetable intake did not influence BMI or other anthropometric measures, such as skinfold or waist to height ratio (WTHR) in children aged 3–6 years [25]. Most reviews in current literature evaluate overall populations that are not specifically low SES. The 2003 National Survey of Children’s Health and 1996 NHANES collected data on children aged 2 to 5 years old and found that children in urban areas were 10.7% obese and 21.8% overweight, while children in rural areas were 12.2% obese and 27.2% overweight [12]. Focusing on interventions in low SES areas, which are often rural, is important to determine which aspects are successful to combat pediatric obesity. In this regard, the latest review published on disadvantaged families in 2014 focused on children ages 0–5 years showed that while the results were more successful to obesity related behaviors for those under 2 years old, specifically, for preschoolers the results were mixed. Of note this review included both studies that took place in the communities and the schools [15]. In addition, a Cochrane systematic review done by Waters et al. (2011) [30] concluded that interventions that involved an emphasis on quality of food in the school curriculum, supportive environments, parental support, and teacher support were effective in reducing adiposity specifically in ages 6–12 years. However, to our knowledge, there are no updated reviews evaluating specifically the effect of school-based intervention in low socioeconomic areas in preschoolers. Therefore, the purpose of this scoping review is to examine the literature to study the effectiveness of the school-based interventions in low socioeconomic settings on adiposity-related outcomes among preschoolers (2–5 years).

## 2. Materials and Methods

The research question that guided this review was: Which are the main factors of the school-based interventions to target childhood obesity or adiposity-related outcomes in preschoolers who live in rural and/or underserved areas? Because of the magnitude of this research question, a scoping review was the best fit to assess the current literature and address future research questions related to this topic. Further, given the variety of study methods on the topic, a scoping review allowed more inclusivity of study types by not assessing the quality of the study itself. This allowed the researchers to assess overall trends and gaps in the literature. The preschool population was defined as 2 to 5 years old in order to eliminate confusion, as this age group may be defined by different ages around the world.

Socioeconomic status is typically conceptualized as the social standing of an individual or group in society in relation to others and is often measured by indicators such as education, occupation, or income or a combination of these [31,32]. We included “rural areas” to point out communities with usually low SES that have health disadvantages such as less access to health care physicians, education, employment which affect health. Research shows that location where people live affects their health and life outcomes. According to the WHO, obesity is more prevalent among poor and socially disadvantaged populations in cities worldwide [33]. “Underserved populations” in the sense of subgroups face barriers to healthcare access (economic, cultural, linguistic) and lack of resources (financial, educational, housing) to make healthy choices in comparison to other socially advantaged populations. The Department of Health and Human Services characterizes underserved, vulnerable, and special communities that include members of minority populations or individuals who have experienced health disparities (e.g., Latino populations, African American populations, new mothers, and women with children etc.) [34]. In addition, Reid et al. (2008) [35], performed a meta-regression that found that there is an increased risk for poor access to health care for individuals who face economic and housing instability.

### 2.1. Search Strategy

The preferred reporting items for systematic reviews and meta-analysis, extension for scoping reviews (PRISMA-ScR) checklist was followed to conduct and report this scoping review. A preliminary literature search was conducted on 19 November 2018 by a graduate student (ML) and a research librarian (HS) to assess the viability of the topic and methodology. The search string was then finalized to include appropriate MeSH (Medical Subject Headings) terms for PubMed/MEDLINE and adjusted the strategy and subject headings accordingly for ERIC (Education Resource Information Center) via EBSCO and Food Science Source via EBSCO. A sampling of the keywords and subject headings used to search are as follows: program; intervention; education; obesity; overweight; BMI; rural; underserved; preschool; child development center. The three electronic databases were searched with no date restriction or other limiters on February 4, 2019. Search strategies are described in Supplementary Table S1.

**Figure 1:**
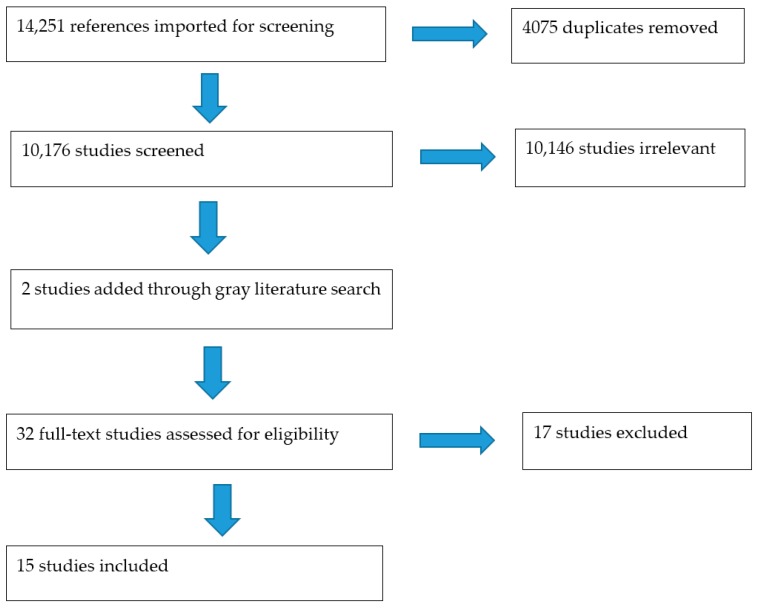
Preferred reporting items for systematic reviews and meta-analysis flow chart.

A gray literature search was performed, after full text screening, by reviewing reference lists of the original 13 selected articles. Figure 1 shows the PRISMA flow chart for further details on total records searched, included/excluded, and for full-text evaluation.

### 2.2. Inclusion and Exclusion Criteria

Covidence software through Cochrane was used to facilitate the review process. All results from the three searches were included for the first round of title/abstract screening. Covidence provided de-duplication of records. Two reviewers (MSP and ML) independently screened for predetermined inclusion/exclusion criteria, which were as follows: Studies that included school-based interventions that reported anthropometric data or obesity outcomes, targeting preschoolers (2 to 5 years old) in rural/low socioeconomic/underserved areas. Articles were excluded if obesity/anthropometric traits or the target population was not mentioned in the abstract, if interventions were community-based, and if the article did not have a translated version in English. A third person (HS) helped with discrepancies.

Covidence was also utilized for full-text screening, which two reviewers (MSP and ML) independently screened for the same criteria as mentioned above. Studies were excluded if they did not include a school-based intervention, if the population age range did not include ages 2–5 years old, if the interventions were not in a rural/low SES setting, and if the studies were not translated to English.

### 2.3. Data Extraction and Synthesis

Final articles are summarized in Table 1, which included population characteristics, methodology, setting, and primary and secondary outcomes. Primary outcomes were specifically related to adiposity-related outcomes (BMI, BMI z-score, waist circumference, skinfold, percent of body fat, etc.) while secondary outcomes included other changes in lifestyle behaviors such as screen time, PA levels, fruit and vegetable intake etc.). Studies were then labeled as “successful”, “unsuccessful,” or “inconclusive” based on significant reductions in primary outcome or adiposity- related outcomes. Unsuccessful interventions were the ones that did not reduce the primary outcomes significantly, and inconclusive interventions were the ones that did not have an effect on the primary outcomes. Table 2 describes the interventions and the results in detail.

## 3. Results

The search identified 14,251 studies that were imported for screening in February 2019 before Covidence de-duplication of the records. Through title and abstract screening, 10,146 studies were irrelevant, and 30 studies were included for full-text screening. Of those 30 records, their reference lists were reviewed for final consideration of any grey literature or additional records to be added. After full-text screening, 13 studies were extracted for study. Additional two studies were included by searching citations from primary references (total studies *n* = 15). See Figure 1: PRISMA and Supplementary Table S1: Search strategy.

Of the final studies, 14 were randomized controlled trials (RCTs) [12,13,17,18,19,20,21,22,24,25,26,27,28,29] while one was a quasi-experimental study [23]. As per our inclusion criteria all the interventions focused on preschoolers, ages 2–5 years old and typically included parental involvement. Most studies were conducted in the United States (*n* = 7) [12,18,19,23,26,27,28], followed by Switzerland (*n* = 2) [17,22], Israel (*n* = 2) [21,29], New Zealand (*n* = 1) [24], Belgium (*n* = 1) [13], Germany (*n* = 1) [25], and France (*n* = 1) [20].

All of the extracted studies were multicomponent interventions that targeted obesity-related outcomes (BMI z-score, waist circumference (WC), skinfold, percent body fat (%BF)). All but one [25] intervention included a PA component [25] (Table 1). Length of the interventions varied with one intervention having a follow-up of 14 weeks [27], one intervention having a follow-up of six months [21], six interventions having a follow-up of one year [17,18,22,25,28,29], six interventions having a follow-up of two years [12,13,19,20,24,26], and one with a follow-up of three years [23]. In addition, the number of children participating in each study ranged from *n* = 18 to *n* = 1926.

### 3.1. Successful Interventions

For this review, a successful intervention was described as one that reduced adiposity-related outcomes including BMI-z score, waist circumference, percent body fat (%BF), etc. Nine out of the fifteen interventions were successful at reducing BMI [13,17,18,19,20,21,22,24,28] (Table 1). All of the interventions were multicomponent, which included nutrition [13,17,18,19,20,21,22,28], PA [13,17,18,19,20,21,22,28], screen time [13,18,19,21,22], and economic emphasis [28]. The adiposity-related outcomes evaluated were waist circumference (WC) [17,22], %BF [17,22], and skinfold measurements [22]. Other secondary outcomes evaluated included fruit and vegetable consumption [13,17,21], water consumption [13,21], sugar-sweetened beverage consumption [13,17,21], food variety, sweet and savory snack consumption [13,17], packed lunch score [21], and sleep [21]. Parents were involved in all of the successful interventions. The length of the studies were six months to three years and the sample size was 146 to 1816 preschoolers. The efficacy of the most successful intervention was conducted in Switzerland. Results found a successfully reduced %BF by 5%, sum of skinfolds by 10%, and WC by 2% [22]. Children were engaged in four 45-min PA sessions/week that targeted aerobic fitness (Table 2). Lessons (*n* = 22) were based on the five recommendations of the Swiss Society of Nutrition: (1) drink water, (2) eat fruits and vegetables, (3) eat regularly, (4) make clever choices, (5) turn your screen off when you eat. Parent involvement and modification in the classroom environment was also targeted as part of the intervention. Despite anthropometric traits, beneficial effects were also shown in PA, nutrition habits, and media use [22].

All successful interventions provided preschoolers with educational nutrition lessons and PA sessions. The majority of successful interventions incorporated themes and/or activities into their lessons. One intervention based in Switzerland focused on themes, such as Spiderman, to reinforce learning [17], while another intervention in France paired games that aimed to increase nutrition knowledge about the food groups, eating balanced meals, and drinking more water [20]. Fitzgibbon et al. (2005) [19] modeled the nutrition lessons after “Go and Grow” foods to teach the food pyramid. Other studies focused on educating preschoolers about healthy choices during the nutrition lesson. PA sessions ranged from 20–45 min long. Parents were involved in each intervention by receiving educational handouts, attending educational sessions, and/or receiving guided PA CDs [13,18,21,28]. Anthropometric measures improved in each intervention. Four out of the nine interventions lowered BMI z-score [13,17,18,20,21]. The intervention conducted by Kaufman-Shriqui et al. (2016) [21] proved to decrease BMI z-score in both the intervention (IArm) and control (CArm) groups. Although there were no anthropometric differences in the results between IArm and CArm, we still consider this to be a successful intervention due to CArm still receiving the PA component.

Two out of the nine successful studies incorporated an economic change, policy change, or environmental change in their intervention [22,23]. An intervention in Switzerland focused on environmental change by changing the layouts of classrooms to promote PA [22]. Changes included adding hammocks, balls, and climbing walls [22]. Lastly, an intervention in California focused on an economic component and encouraged parents to buy budget-friendly nutritious foods. Modeled after WIC (Special Supplemental Nutrition Program for low income Women, Infants, and Children, a US federal assistant program) program families received $25 vouchers that could only be used to buy fruits and vegetables [23].

### 3.2. Inconvlusive Interventions

Inconclusive interventions were described as the ones that did not significantly reduce anthropometric measures. One study proved to be null with no positive outcomes [26]. Six out of fifteen interventions failed to reduce BMI and/or BMI z-score [12,25,26,27,28,29]. All of the interventions were multicomponent, which included nutrition, PA, media use, and sleep. The studies evaluated the association of the intervention with different obesity-related measures, including BMI z-score, %BF, WTHR, and skinfold. Other outcomes evaluated included aerobic fitness, screen time, and consumption of water, milk, vegetables, fruits, soft drinks, sweet, dietary fat, fiber, and savory snacks. Parents were involved in all of the six inconclusive interventions. The length of the studies were fourteen weeks to two years and the sample size was 377 to 1926 preschoolers.

All interventions provided preschoolers with educational lessons or informational sessions about proper nutrition and PA. Each had themes or activities incorporated into the interventions. Three of the studies incorporated puppets or pirate dolls to reinforce learning [25,26,27]. Three studies utilized scripted exercise CDs, which were translated into multiple languages to be accessible to parents, that led the children in a short PA activity [27,28,29]. Other studies incorporated activities that were aimed at trying new healthy foods or making healthy snacks in the classroom [25,28]. Although a reduction in BMI did not occur in these studies, other positive outcomes were reported. Bock et al. (2011) [25] reported an increase in vegetable intake (0.027) [25]. Fitzgibbon et al. (2006) [26] did not report any significant conclusions, while in a later study the same author (Fitzgibbon et al., 2011) [27] reported a decreased screen time (–27.8 min/day (–55.1 to −0.5), *p* = 0.05) and increased moderate to vigorous activity (MVPA) compared to the control group (moderate, 4.78 min/day (0.10 to 9.45); *p* = 0.05 and vigorous, 2.83 min/day (−1.8 to 66.9), *p* = 0.03). An intervention in Chicago reported that the intervention group increased intake in whole fruit (*p* = 0.02), total fruit (*p* = 0.003), and whole grains (*p* = 0.02) compared to control group [28]. Lastly, the intervention in Israel, modeled after the Israeli Ministry of Education program called “It Fits Me,” indicated an increase in nutrition and PA knowledge and preferences (*p* < 0.05) in the intervention group [29].

### 3.3. Theoretical Frameworks

Six out of the fifteen studies based their interventions on theoretical models [13,18,23,25,26,27] and of those six, three were successful interventions [13,18,23]. Theoretical models mentioned were theory of social learning, social cognitive theory, self-determination theory, socioecological model of health, and health belief model. Bock et al. (2011) [25] based the parental component of the intervention on the theory of social learning. The theory of social learning in the context of learning food behaviors suggests that children learn by imitating their parents and peers. Therefore, including parents in the intervention is important when changing child behavior [25]. Coen et al. (2011) [13] based their intervention on the socioecological model of health, which emphasizes on the child while considering the several layers of interaction around them (parents, friends, media etc.). Fitzgibbon et al. (2006), (2011), and (2013) based their interventions on social cognitive theory and self-determination theory. Fitzgibbon et al. (2013) [18] also included the health belief model. Sadeghi et al. (2016) [23] based their interventions on the social cognitive theory and the health belief model.

## 4. Discussion

This scoping review aimed to examine the effectiveness of school-based interventions that targeted pediatric obesity in preschool children (ages 2–5) in low-SES areas. A total of 15 interventions met the inclusion criteria: Studies that included school-based interventions; reported obesity related/anthropometric outcomes; targeting preschoolers (2 to 5 years old) in rural/low socioeconomic/underserved areas. Nine out of the fifteen studies were considered to be successful while five studies were inconclusive, and one was null. Successful interventions demonstrated significant reductions in BMI-related anthropometric measures. Both successful and inconclusive interventions included PA and nutrition components and also involved parents as part of the targeted intervention.

Reviews and systematic reviews on obesity prevention that include general population and a broad range of children ages (0–14 years) and settings [36,37,38,39] are in line with the successful interventions that we reported. Interventions that promote healthy eating in preschoolers thrive when paired with other components, such as PA [10]. Additionally, the two components that all successful interventions had in common are parental and child involvement. Intervention activities geared toward the preschoolers were most successful when the children were engaged in interactive learning, such as taste testing, playing games, and singing songs [10,21]. Parent sessions were focused on nutrition, PA education, and how healthy food shopping can be budget-friendly [13,18,21,22]. While successful interventions varied in duration, they all aimed to increase nutrition knowledge, PA, and decrease obesity-related anthropometric measures [10,12,13,17,18,19,20,21,22,23,24].

Our findings support current literature in that successful obesity-prevention interventions for preschoolers are multicomponent [10,15]. Parental involvement in successful interventions is crucial in order to intervene in pediatric obesity [16,40]. Focusing on parents in addition to their preschoolers is important because their attitudes, beliefs, and behaviors contribute to weight gain in their child [41]. These behaviors, such as feeding styles, role modeling, and sharing nutritional knowledge with their children have been associated with their children’s weight, eating habits, and PA levels [36,37,41,42]. Implementing parental involvement in such interventions may encourage children to try new foods and model healthy eating behaviors after their parents [36,41,42]. The importance of parental involvement was also supported by Verjans-Janssen et al. (2018) [16] by suggesting that the parental component should be the primary focus of such interventions. Thus, focusing on parent–child bonding may increase parents’ interest in participating. Implementing interventions in more diverse populations will allow for a better understanding of how to implement a family-based component [38]. Although all the interventions evaluated in this scoping review were multicomponent, the intensity and the delivery mode as well as the content may affect the success of the intervention. For example, Burgi et al. (2012) [17] included a reward system in class with stickers that may keep children’s motivation. Others included well-known characters “Spiderman” to engage kids to change behaviors.

Theoretical-based interventions were scarce as has been mentioned previously. Only six interventions included theoretical models [13,18,23,25,26,27] and three were successful in reducing obesity outcomes [13,18,23]. Future studies should consider elaborating their interventions based on a theoretical model.

Affordability and food costs may be a barrier for families living in rural/low SES areas. Economic change, policy change, and environmental change might be an important point to take into account when implementing obesity school-based interventions, in particular, in low SES settings, however there are not enough studies evaluating these strategies. Research shows that a majority of people associate eating healthy with being expensive; however, a systematic review and meta-analysis by Rao et al. (2013) [43] evaluated studies from 10 different countries. Significant monetary differences occurred in meats/protein with healthier options costing $0.47/200 kcal and $0.29/serving more than less healthy meats/proteins. Price differences were less significant in healthier grain and dairy options ($0.03/serving and −$0.004/serving, respectively). Thus, eating a healthy and balanced diet cost $1.48/day and $1.54/2000 kcal more than a less healthy diet [16]. Although, McDermott et al. (2010) [44] specifically analyzed low-income, single-parent populations and the monetary difference between food shopping at a supermarket and eating primarily fast-food (convenience diet). Using cost-per-calorie analysis, convenience diets accounted for 37% of income, while a healthy diet accounted for only 18% of income. Sadeghi et al. (2016) [23] provided monetary incentives in the form of a voucher for families to spend on fruit and vegetables in addition to “family nights” when nutrition lessons would be held. However, more research is needed to determine if vouchers are a successful component to incentivize healthy eating. Educating parents on how to plan a long term healthy and sustainable menu within a budget may help them to have healthy choices and thus, help decrease obesity outcomes.

Low SES populations are highly specific and may not adhere to a general obesity intervention. These populations along with minority youth are at an increased risk for obesity, type 2 diabetes, and other comorbidities [45,46,47]. For example, Latinos face barriers that non-minority populations do not, such as limited access to health insurance, language and cultural barriers [47,48]. Additionally, a popular perception in the Latino population is that the heavier the child, the healthier [49,50]. Thus, interventions targeting low SES and minority youths require tailored interventions specific to the population. To demonstrate the need for tailored interventions, Falbe et al. (2015) [47] conducted a 10-week active and healthy families (AHF) intervention that targeted Latino youth aged 5 to 12 years old. The intervention consisted of five 2-h group medical appointments, which focused on topics such as, obesity, nutrition labels, healthy snacks, PA, emotional eating, portion sizes, fast food, parenting, stress, and immigration. PA sessions were implemented during parent–physician discussions. Results showed a decrease in BMI in the AHF group (−0.50 kg/m^2^, CI (−1.28, −0.27)) [47]. Tailoring interventions to the culture are also supported by a systematic review by Tovar et al. (2014) [51]. Successful interventions in youth had a cultural focus, which facilitated community engagement and parental involvement. Based on the results from this scoping review, studies in this regard are still scarce, and more research is needed in order to determine which components have the best effect in low SES and minority youth populations.

Strengths of this scoping review include a unique focus on preschoolers (ages 2–5), low SES populations, and school-based interventions. Two researchers were used to screen all studies through a systematic search process, and disagreements were decided by a third person (HS). Limitations include not including search terms that represented different languages. We did not limit to English specifically, but that is inherent in our search strategies. There were also limited studies for a global focus, which can be due to the language limitations. Additionally, defining these programs was difficult and may be due to different definitions (i.e., preschool) throughout the world, but it was also limited to the databases we searched. Lastly, there is no way to be all inclusive in a gray literature search and due to a small effect size of successful interventions, we do not have enough successful interventions to make a conclusion.

## 5. Conclusions

Based on our results, multicomponent school-based interventions targeting low SES preschool children should include a nutrition and PA component and involve parents as agents of change. Although there is not an outstanding component that can discriminate successful vs. inconclusive interventions, future studies should focus on culturally tailored interventions, consider exploring and including economic change (know how to be healthy within a friendly budget), school environment, and assess motivation and engagement of children to achieve healthy behaviors that translate into obesity reduction. In addition, the interventions may be more effective if hands-on learning were involved by integrating a structured food education framework. However, so far, the literature is still limited, and the evidence is scarce, and more research with rigorous evaluation and high-quality study designs that focus on disadvantage/undeserved/low SES population are needed to establish key components to reduce pediatric obesity in low SES settings.

## Figures and Tables

**Table 1:** Summary of studies evaluating school-based interventions among preschoolers in low socioeconomic status (SES) settings to tackle pediatric obesity.

Reference	Country	Study Design	Sample Size	Population (age)	Follow-Up	Components	Intervention Target Population	Outcomes
**Successful interventions**								
Coen et al. 2011 [13]	Belgium	Cluster-RCT in six communities paired by SES 31 schools Intervention group: 18 Control group: 13	1589	Ages 3–6 years old	2 years	Nutrition, PA, Screen time,	Children, Community, Schools (teachers), Parents	Primary outcome: BMI z-score
Burgi et al. 2012 [17]	Switzerland	Cluster-RCT 40 preschools Intervention: 20 schools Control: 20 schools	652	Socially disadvantaged—preschoolers (72% migrant, 38% low EL for parents), average age 5.2 ± 0.6 years	1 year	PA, Nutrition, Sleep, Media use,	Children, Teachers, Parents	Primary outcomes: %BF, WC Secondary outcome: Aerobic fitness
Fitzgibbon et al. 2013 [18]	Chicago, United States	Cluster-RCT 4 schools randomized into WCI (intervention, 2 schools) or GHI (control, 2 schools)	146	Hispanic children (low income minority), 3–5 years old	14 weeks intervention. 1 year follow-up.	Nutrition, PA	Children, Parents	Primary outcomes: BMI, BMI z-score
Fitzgibbon et al. 2005 [19]	Chicago, United States	RCT	420	Average age: 4 years	2 years	Nutrition, PA, Media Use,	Children, Parents	Primary outcome: BMI Secondary outcome: Calories from saturated fat
Jouret et al. 2009 [20]	France	RCT 79 kindergarten (schools) randomized into two intervention groups: EPIPOI-1 group EPIPOI-2 group. Control data retrieved by the Division of School Health	1780	3–4 years old, 5–6 years old	2 years	Nutrition, PA, Media Use,	Children, Teachers, Parents, Physicians at the school	Primary outcome: BMI z-score
Kaufman-Shriqui et al. 2016 [21]	Israel	RCT 16 schools randomized into intervention group (11 schools; IArm) control group (5 schools; CArm)	258	Low SES school children, ages 4–7 years	6 months	Nutrition, PA	Children, Mothers, Teachers to make healthy choices in a SES area.	Primary outcome: BMI z-score Secondary outcomes: food variety, habitual water drinking, fruit and vegetable intake, sugar sweetened beverage intake, overall packed lunch score, screen time
Puder et al. 2011 [22]	Switzerland	RCT single-blinded 40 preschools; randomization of classes 1:1	652	4–6 years old in high migrant populations	1 school year	PA, Nutrition, Media use, Sleep, Environment,	Children, Parents, Teachers, School Curriculum, Class environment	Primary outcomes: %BF, sum of skinfolds, WC. Secondary outcomes: motor agility, PA, nutrition habits, media use, aerobic fitness
Sadeghi et al. 2016 [23]	California, United States	Quasi-experimental intervention Intervention and comparison community	422	Children from Mexican origin communities, 3–8 years old	3 years	Nutrition, PA, Economic,	Children, Community, Parents, Schools	Primary outcomes: BMI, skin fold thickness, WC
Rush et al. 2012 [24]	New Zealand	RCT 124 schools were randomized with stratification by SES and rurality to either the intervention (*n* = 62) or control group (*n* = 62)	1926	5 years old	2 years	PA, nutrition	Children, Teachers, Graduates or PE teachers, Parents, Local community	Primary outcomes: %BF
**Inconclusive Interventions**								
Davis et al. 2016 [12]	New Mexico, United States	Cluster RCT Intervention group: 8 centers Comparison group: 8 centers	1816	American Indian and Hispanics, 4 years of age or younger	2 school years	Nutrition, PA, Behavior, change, Policy change	Children, Teachers, Health care providers, Food service staff, Parents	Primary outcomes: BMI
Bock et al. 2011 [25]	Germany	RCT Children were randomized into intervention group (*n* = 10) or waiting-list control arm (*n* = 8). Control received same intervention six months later	377	3–6 years old	1 year	Nutrition	Children, Parents	Secondary outcomes: fruit and vegetable intake
Fitzgibbon et al. 2011 [27]	Chicago, United States	RCT 18 schools randomized into either TD-WCI intervention or TD-GHI control groups	618	African American children, 3–5 years old	14 weeks	PA, Nutrition	Children, Teachers, Parents	Secondary outcomes: PA, screen time
Kong et al. 2016 [28]	Chicago, United States	RCT 18 schools randomized into either TD-WCI intervention or TD-GHI control groups	618	Primarily African American children, 3–5 years old	1 year	Nutrition, PA	Children, Parents, Teachers	Secondary outcomes: diet quality, screen time
Nemet et al. 2011 [29]	Israel	RCT	725	3.8–6.8 years old	1 school year	Nutrition, PA	Children, Parents	Secondary outcomes: PA, nutrition knowledge, fitness improvement
**Null Interventions**								
Fitzgibbon et al. 2006 [26]	Chicago, United States	RCT 12 schools randomized into intervention or control group	420	Latino children, 3–5 years old	2 years	Nutrition, PA	Children, Parents	No outcomes

RCT: randomized controlled trial; PA: physical activity; SES: socioeconomic status; BMI: body mass index; EL: education level; WCI: weight control intervention; GHI: general health intervention; TD-WCI: teacher-delivered weight control intervention group; TD-GHI: teacher-delivered general health intervention control group; WC: waist circumference; BF: body fat; EPIPOI-1: “basic strategy”, which gave an overview of weight and health; EPIPOI-2: basic strategy and reinforced strategy (education program to target healthy nutrition, PA, and less screen time).

**Table 2:** Detailed descriptions of the school-based interventions among preschoolers (ages 2–5) in low socioeconomic settings.

Reference	Intervention/Study Name ^1^	Theoretical Framework	Detailed Description of Intervention	Detailed Description of Results
**Successful interventions**				
Coen et al. 2011 [13]	POP (Prevention of Overweight among Pre-school and school children)	Socioecological model of health	**Community:** Two intervention meetings were held in each community that provided brochures and information about local health topics. **Schools:** Schools received seven modules that described how to implement the intervention effectively. Schools were advised to designate five “healthy weeks” per intervention year with an hour of class time each week for extracurricular healthy activities (i.e., tasting vegetables), improve their playground, revise snack policies, and communicate/distribute educational materials to parents. **Parents:** Parents were given a poster with target messages and key points to help them encourage healthy behavior with their children. They were also given five letters describing, in detail, the intervention topics with tips, tricks, and recipes that they can try with their children. Lastly, they received a report of their child’s food intake, such as fruits and vegetable intake, as well as PA and screen time with ways they can make improvements.	No significant effects in BMI z-score occurred in the total sample, but there was a statistically significant decrease of 0.11 in BMI z-score in the low SES intervention group (*p* = 0.01) compared to their pair low SES control group (BMI increased by 0.04 (F = 6.26, *p* = 0.01)). No significant effects on consumption of water, milk, vegetables, soft drinks, fruits, sweet and savory snacks, PA, or screen time.
Burgi et al. 2012 [17]	Ballabeina study	N/A	**Culturally tailored intervention** **Children:** PA program 4 days/week for 45 min/session. Sessions aimed to increase aerobic fitness and coordination. Each had themes, such as Spiderman. Health promoters and teachers led the sessions. Twenty-two lessons on nutrition, screen time, and sleep in addition to healthy snacks were given to the students. **Teachers:** Attended two three-hour workshops to learn the content they will be teaching and share experiences. They received hands on training from health promoters. **Parents:** Attended three informative sessions discussing PA promotion, nutrition, screen time, and sleep. Informative brochures, worksheets, and exercises were handed out that preschoolers brought home. These handouts were translated in 10 different languages.	**Children of migrant/non-migrant parents:** Both migrant/non-migrant preschoolers benefitted from the intervention. Both groups had a significant change in %BF (−1.42%; *p* = 0.013 for non-migrants; −1.14%; *p* = 0.015 for migrants) and waist circumference (−0.86 cm, *p* = 0.01 for non-migrants; −1.02 cm; *p* = 0.003 for migrants). Aerobic fitness change was more significant in non-migrant preschoolers compared to migrant preschoolers, while agility changes were only significant in migrant groups. Specifically, preschoolers of African descent improved their aerobic fitness the most (*p* = 0.02). **Children of low EL parents:** Low EL children did not benefit from the intervention as much as middle/high EL parents’ children (differences were only borderline significant: *p* for interaction ≥0.2 for adiposity and agility; *p* for interaction = 0.06 for aerobic fitness) except for waist circumference (middle/high EL *p* = 0.004; low EL *p* = 0.017). No significant effects in the BMI z-score occurred in the total sample. No effects between SES, BMI, and behavior.
Fitzgibbon et al. 2013 [18]	Hip Hop to Health	Social cognitive theory, self-determination theory, health belief model	14-week intervention led by bilingual educators. **Children:** Intervention included 20 min nutrition lesson/20 min aerobic PA. To enhance the original intervention, a Spanish language CD was handed out to supplement the material. Control group received one intervention/week (20 min) that consisted of dental health, calling 911, and seat belt safety. **Parents:** Parent intervention still included educational handouts on nutrition and PA geared toward lower income Hispanics. Parents of preschoolers received 90-min classes (60 min discussion on nutrition and 30-min PA time)	BMI and BMI z-score were significantly lower in both groups after one-year follow-up. The difference between groups was statistically significant for BMI change adjusted for different covariates including BMI at baseline (0.22 kg/m^2^ (0.02–0.041); *p* < 0.005) MVPA and accelerometer counts/min were higher in intervention group than in control group, but the difference was not statistically significant. No difference between screen time in intervention (3.4 (0.5) h/day at follow-up) and control group (3.3 (0.5) h/day at follow-up). No significant differences in diet in intervention or control group.
Fitzgibbon et al. 2005 [19]	Hip Hop to Health Jr.	N/A	14-week intervention led by teachers **Children:** Intervention included a 20-min lesson that focused on healthy eating or an exercise concept, followed by 20 min of PA. The lessons incorporated hand puppets to teach the food pyramid, “go and grow” foods and reducing tv viewing. PA sessions included a 5-min warm-up, 10 min of aerobic activity, and 5 min of warm-down. Children in the control group received a 20-min lesson for 14 weeks on general health concepts. **Parents:** Weekly newsletters were sent home that addressed the children’s curriculum and included a homework assignment. Assignments were made to reinforce concepts in the newsletter. $5 grocery coupon was given for each assignment they finished. EX: Keep track of your child’s fruit and vegetable intake for one week and think of ways you can increase your family’s fruit and vegetable intake.	Intervention group had significant smaller increases in BMI than the control group at the one-year follow up (CI −0.91 to −0.14) and two-year follow-up (CI −0.98 to −0.10). Intervention group also demonstrated a difference in calories from saturated fat at the one-year follow-up compared to the control group (11.6% vs. 12.8%; *p* = 0.002).
Jouret et al. 2009 [20]	Epidemiologie et prevention de l’obesite’ infantile, EPIPOI	N/A	**Children:** Three groups that children were put into: EPIPOI-1, EPIPOI-2, and control. EPIPOI-1 that followed “basic strategy”, which gave an overview of weight and health; and EPIPOI-2 received basic strategy and reinforced strategy (education program to target healthy nutrition, PA, and less screen time). Ten 20-min sessions (5 sessions/year) were given to the children. They incorporated activities/games that aimed to gain knowledge about the food groups, eating balanced meals, having breakfast, limiting sugar-sweetened beverages, drinking water, and increasing PA. Kids received a cassette and story book to reinforce the messages. **Parents:** Parents were provided information to enhance what the students were learning in the classroom.	BMI z-score were lower in both intervention (change EPIPOI-1: 0.35 (0.19; 1.04); change EPIPOI-2: 0.50 (0.17; 1.01) groups compared to control (change in control: 1.35 (0.57; 1.82); EPIPOI-1 vs. control *p* < 0.001; EPIPOI-2 vs. control *p* < 0.001) in underprivileged areas but no difference between intervention groups.
Kaufman-Shriqui et al. 2016 [21]	Intervention in Israel	N/A	IArm: received nutrition intervention and PA Carm: PA only. **Children:** All lessons were delivered by clinical dietitians, PA teachers, and economists that specialize with budget planning. Main themes were discussing healthy affordable nutrition, nutrition recommendations (increase fruits/vegetables, water intake, legumes, and decrease high fat, sugar, and sweet beverages), and how to pack healthy lunches. Increasing PA. Mothers and children received lessons structured around these themes in addition to children receiving 10, 45-min weekly lessons given by dietitians. Lectures, stories, games, and songs were utilized to enforce learning. All children received 15 weeks of PA for 45 min/session. **Parents:** Parents in intervention group attended meetings that were centered around low SES nutrition options and how to pack healthy lunches. IArm teachers received special training to enhance the intervention (teachers were trained to compliment students who brought in healthy lunches).	BMI z-score decreased in the entire study population by 0.1 (*p* = 0.003). Intervention group showed a greater increase in food variety, habitual water drinking, fruit and vegetable intake and a decrease in sugar-sweetened beverage. Overall packed lunch score improved significantly in intervention group (decrease in non-healthy snacks/sweetened beverages, and an increase in fruits and vegetables) (*p* < 0.001). Consumption of water increased in Iarm group (*p* = 0.02). Average PA time decreased more from baseline in control group than in intervention group (−0.42 ± 0.01 h (−18.4%), −0.21 ± 0.01 h (−8.4%)). Mean screen time increased in both groups but significantly more in control group. Nutrition knowledge increased significantly in intervention group by 16% at month three and 39% at month six.
Puder et al. 2011 [22]	Ballabeina study	N/A	**Culturally tailored intervention** **Children:** Participated in four 45-min PA sessions/week, which targeted aerobic fitness by playing games (taught by exercise physiologist). Health promoters (exercise physiologists) taught one PA session/week in the beginning and then reduced to twice/month while teachers taught the remaining sessions. Twenty-two lessons on nutrition, media use, and sleep were taught. The lessons were based on the five recommendations of the Swiss Society of Nutrition (drink water, eat fruits and vegetables, eat regularly, make clever choices, and turn your screen off when you eat). Children received funny cards about PA or nutrition every other week to take home. CDs were also created to support PA time. Healthy snacks were promoted as well as stickers were posted in the classroom that enhanced the intervention. **Teachers:** Attended two workshops before the intervention started. They received all materials for the lessons in advance. They learned how to lead the PA session by hands-on training by the health promoters in advance. **Parents:** Attended three meetings that discussed the importance of PA, nutrition, screen time, and sleep. Materials to enhance the topics were provided, such as brochures. Handouts were offered in 10 different languages. **Environmental factors:** School environment was adapted to promote PA. Movement corners in classrooms were provided, such as hammocks, balls, cords, climbing walls. A poster of the “Ballabeina track” was hung in the classroom that included the themes and stickers to track their progress. **Control group:** no intervention. One 45-min PA lesson once/week plus one discussion for the parents.	No different in BMI compared to controls (*p* = 0.31), but intervention group showed a reduction by 5% in BF (−1.1%, −2.0 to −0.2; *p* = 0.02),10% in sum of skinfolds (−2.78 mm (−4.35 to −1.2)), and 2% waist circumference F, (−1.0 cm, −1.6 to −0.4; *p* = 0.001). Intervention group also demonstrated improved motor agility (*p* = 0.004), but not improved balance. Beneficial effects were also seen in PA, nutrition habits, and media use. Significant increase in aerobic fitness in intervention group compared to the control group (*p* = 0.01).
Sadeghi et al. 2016 [23]	Ninos Sanos, Familia Sana (NSFS) intervention	Social cognitive theory, health belief model	**Nutrition:** Education was delivered to parents during “family nights” and to the children during school hours. Nutrition lessons consisted of skill building and parenting skills through interactive methods. A nutrition educator delivered the materials to the parents and planned a different topic each month. Ten sessions were offered, but only 5 were mandatory for parents. Teachers delivered the nutrition lessons to the children during school time. The lessons were based on UCCE curriculum, which included “Reading across MyPyramid.” **PA:** PA sessions were integrated with sport play and active recreation for kids (SPARK) in addition to the original PE curriculum. Originally teachers instructed PE, but with the intervention, the school hired a PE teacher. Teachers were provided with SPARK materials (CDs, binders, equipment). Lessons lasted 20–30 min. Preschool teachers gave lessons daily. **Economic:** Designed to encourage nutritious foods that are budget friendly. Modeled after WIC. Families received $25 vouchers that could only be used to purchase fruits and vegetables.	Girls: triceps skin fold thickness increased significantly in control than intervention (*p* = 0.05). No other significant change within the first year of intervention. Intervention was successful as reducing the change in BMI from baseline in boys who were obese only (ß-coefficient = −1.94, *p* = 0.05). Boys in intervention group demonstrated a small decrease in waist circumference (ß-coefficient = −5.2, *p* = 0.04) compared to the comparison group.
Rush et al. 2012 [24]	Project Energize	N/A	**Children:** Classes included games, fitness activities, ball activities, and sports-related games to keep the kids moving. They also promoted lunchtime games, bike days, and leadership training so that students could lead a PA session before and after school. Healthy-eating initiatives were also enforced, such as removing pastry-based pies and big cookies in the schools and adding fruit, low-fat yogurt, and filled rolls. Fund raising included selling water and non-food items instead of baked goods and sugary sweets. Teachers also taught lessons, such as why drinking water/milk instead of sugary drinks are important and how to pack healthy lunches/snacks on a budget. **Parents:** A school newsletter was sent out every week with a “nutrition nugget” that was featured. They included 30 smart swaps (replacing instant noodles with baked beans etc.) and discussing what are “every day,” “sometimes”, and “occasional” foods. A home-school link program was established for parents to attend 45-min sessions that highlighted healthy dietary choices. They received laminated cards and magnets that reinforced nutrition goals. Assistance was provided to parents, teachers, and local community by organizing activities with dietitians and professional development leaders to discuss nutrition. Control group did not receive resources.	BF% standard deviation score was improved in 5–7-year old (effect of the intervention −0.14 (−0.26, −0.01), *p* = 0.03). However, the statistically significant difference disappears after adjusting by cluster. The effects of the intervention occurred more in rural and schools with higher SES (although they were not statistically significant; *p* = 0.08).
**Inconclusive interventions**				
Davis et al. 2016 [12]	Child Health Initiative for Lifelong Eating and Exercise (CHILE) intervention	N/A	Intervention occurred at 16 different Head Start centers in rural American Indian and Hispanic communities that focused on six components: At least 30 min of PA daily and time to taste new vegetables, development training for staff, integrating policy and behavior change with food preparation staff, take home materials for the family, grocery store component to increase availability of healthier food options; and ask local healthcare providers to emphasize healthy eating and OA during routine patient visits.	At the end of the intervention period, no significant difference in BMI in the intervention and comparison groups were found. Values were almost the same.
Bock et al. 2011 [25]	‘Come aboard the health boat’ intervention in Germany	Theory of social learning	Nutrition experts gave the intervention in 2-h sessions, 15 times. These sessions occurred once a week over six months during preschool hours. Five of the sessions involved parents, which focused on the nutritional needs of their children. Activities conducted with the children included eating meals together as a class, teachers, and parents, addressing healthy drinking behaviors, playing with pirate dolls who enjoyed eating fruits and vegetables, and trying new healthy foods.	No significant effects on BMI, WTHR, or skinfold. In the subgroups of overweight and obese children (n 18 with pre- and post-term measurements), bivariate analyses also showed no significant effect of the intervention on BMI, WTHR and skinfold sum (data was not shown). There was a significant difference in the pre and post intervention fruit and vegetable intake (increase from 0.17 at baseline on a 6-point ordinal scale to 0.22 points post intervention, *p* < 0.001 and *p* < 0.05 respectively). Post intervention produced a change in vegetable consumption (*p* = 0.027). No significant effects on water intake and high-energy drinks.
Fitzgibbon et al. 2011 [27]	Hip-Hop to Health Jr. obesity prevention	Social cognitive theory, self-determination theory	**Children:** Childhood educators taught either a 14-week PA and nutrition intervention program (twice per week) or a general health. Each intervention session had a theme that included a 20-min lesson on PA and nutrition and a 20 min physical activity session. Lessons included the pyramid puppets, which represented the seven different food groups. PA sessions included songs and raps that had scripted exercise routines. **Parents:** Parents received educational handouts that corresponded with their child’s health homework. $5 incentive was given to parents for each homework assignment completed and returned. They were also given the same PA CD that the teachers play for the preschoolers, so they could implement PA at home.	No significant differences between groups in BMI post intervention. No difference in TV watching between groups and in diet (energy intake, dietary fat, consumption of fruits or vegetables). Preschoolers in the intervention group engaged in more moderate and vigorous PA than preschoolers in control school (*p* = 0.02) and had less total screen time than control group (*p* = 0.01).
Kong et al. 2016 [28]	Hip-Hop to Health Jr.	N/A	Eighteen schools were randomized into two groups: a teacher-delivered intervention curriculum or a teacher-delivered general health curriculum. **Children:** Intervention curriculum targeted PA, diet, and decreased TV time. Delivered two times/week for 14 weeks. Sessions were theme based with a 20-min lesson on healthy eating or PA and a 20-min session with a PA component. CDs were given out to families and also used in the classroom. In food-themed lessons, the children helped prepare snacks and took part in food tastings. The difference between other Hip-Hop to Health interventions was that there was a modification to lesson frequency. Teachers were allowed to present the lessons twice/week instead of thrice/week. The third lesson per week was now optional and reinforced the content learned during the week instead of introducing new material. The PA portion was led by the teacher with the guidance of the CD. **Parents:** Parents still had the same home assignments (completing and turning in homework assignments with kids and receiving the same PA CD).	BMI z-score change was not significantly different after intervention between the two groups (*p* = 0.83). TV viewing time did not change post intervention. Fruit consumption and overall diet quality in intervention group remained the same but decreased in control group. Significant between group differences include whole fruit (*p* = 0.02), total fruit (*p* = 0.003), SoFAAS (*p* = 0.02), and whole grains (*p* = 0.02). Between group difference for fruit/day was 0.35 servings/day (SE = 0.14, *p* = 0.02). Although, HEI-2005 scores worsened in the control group, which suggest that early intervention might improve diet quality over a longer period of time. Screen time was reduced in intervention group but was not sustained throughout the year follow-up. A limitation to the intervention was that teachers did not deliver the intervention with as much intensity as the research team. Specifically, the PA portion was not consistent because the teachers themselves were not as active and it was a challenge to teach PA.
Nemet et al. 2011 [29]	“It fits me” intervention in Israel	N/A	Intervention lasted one school-year. **Children:** Nutrition lessons were modeled after the nutrition program “It Fits Me” from the Israeli Ministry of Education. Topics taught included healthy food choices, vitamins, food preparation, cooking methods, and fast food vs. home-cooked meals. Children were then asked to talk about what they learned with their parents. PA sessions were 45 min/day, six days/week. Five sessions were taught by preschool teachers and one session was taught by a professional youth coach. Children were then encouraged to increase PA after school and decrease sedentary activities. CD’s with songs were often used to enhance the nutrition and PA lessons. Control group continued the regular school curriculum. **Parents:** Monthly flyers were sent home to reinforce material, which also encouraged discussion time with their children about what they have been learning in class. Parents and their children attended two “healthy day festivals” that focused on the intervention themes. Parents and children participated in activities and parents also had the option to attend lectures by the study team.	No differences in BMI, but nutrition knowledge and preferences, PA knowledge and preferences, and fitness improvement all increased (*p* < 0.05). At the end of the school year there was a larger decrease in overweight children in the intervention group (−31.9%) compared to the control group (−17.5%).
**Null interventions**				
Fitzgibbon et al. 2006 [26]	Hip-Hop to Health Jr.	Social cognitive theory, self-determination theory	A 14-week (3 sessions/week) intervention consisting of diet and PA. Each session included 20 min of food pyramid education with hand puppets and 20 min of aerobic activity. The intervention targeted decreased fat intake, increase fruit/vegetable intake, decreased sedentary behavior, and increased PA. Delivered in Spanish and English. Parents received weekly educational letters that enhanced the child’s curriculum and homework assignments that were turned in for monetary compensation.	No differences in BMI and BMI z-score from baseline to post intervention (*p* = 0.56). No significant differences between saturated fat/fiber consumption. No significant differences in PA or screen time.

^1^ For those studies without a “name” for the intervention we included only the location where the intervention took place. CHILE: child health initiative for lifelong eating and exercise; PA: physical activity; BMI: body mass index; WTHR: waist to height ratio; %BF: percent body fat; MVPA: moderate to vigorous physical activity; WIC: women, infants, and children; PE: physical education; SES: socioeconomic status; SoFAAS: calories from solid fat, alcohol, and added sugar.

## Supplementary Materials

The following are available online at https://www.mdpi.com/2072-6643/11/7/1518/s1, Table S1: Search Strategy.
