# Supplementary material for: School-Based Interventions in Low Socioeconomic Settings to Reduce Obesity Outcomes among Preschoolers: A Scoping Review
Source: Nutrients. 2019 Jul 4;11(7):1518. doi: 10.3390/nu11071518 (PMC6683276; doi:10.3390/nu11071518)
Supplement: Supplementary file 1 [file nutrients-11-01518-s001.pdf]

Supplementary Table

Table S1. Search strategy.

Source	PubMed/Medline	Food Science Source	ERIC	Gray Literature Search
**Platform details**	PubMed	EBSCO	EBSCO	PubMed
**Search Limits applied**	None	None	None	None
**Final Search String(s)**	(program* OR intervent* OR educat* OR prevent* OR promot*)<br><br>AND ("obesity"[MeSH Terms] OR "obesity"[tiab] OR "obese"[tiab] OR "exercise"[MeSH Terms] OR "exercise"[tiab] OR fitness[tiab] OR "physical activity"[tiab] OR "overweight"[MeSH Terms] OR "overweight"[tiab] OR "over weight"[tiab] OR "body mass index"[tiab] OR BMI[tiab] OR "weights and measures"[MeSH Terms] OR "weight"[tiab] OR "body weight"[MeSH Terms] OR "body weight"[tiab] OR "waist circumference"[tiab] OR "diet"[MeSH Terms] OR "diet"[tiab] OR sedentary[tiab] OR "health"[MeSH Terms] OR "health"[tiab] OR "wellness"[tiab] OR "nutritional status"[MeSH Terms] OR "nutritional status"[tiab] OR "nutrition"[tiab] OR "nutritional sciences"[MeSH Terms] OR "nutritional sciences"[tiab] OR "body fat"[tiab] OR "feeding behavior"[tiab] OR "health behavior"[tiab] OR "life style"[tiab] OR "physical education and training"[tiab] OR "motor activity"[tiab] OR "movement"[MeSH Terms] OR "movement"[tiab] OR "Child	(program* OR intervent* OR educat* OR prevent* OR promot*)<br><br>AND (obes* OR exercise OR fitness OR "physical activity" OR overweight OR "over weight" OR over-weight OR "body mass index" OR BMI OR weight OR "waist circumference" OR diet OR sedentary OR health OR wellness OR nutrition OR nutritional OR "body fat" OR “feeding behavior” OR “health behavior” OR food OR fruit OR drink OR vegetable OR “physical education” OR “motor activity” OR movement OR “life style” OR “physical health” OR “body composition”)	Same as Food Science Source	Searched citations from the original 13 primary sources. Eligible studies were found from two original citations: Fitzgibbon et al. 2011 and Burgi et al. 2012

	Nutrition Sciences"[tiab] OR "drinking"[MeSH Terms] OR "drinking"[tiab] OR "food"[MeSH Terms] OR "food"[tiab] OR "fruit"[MeSH Terms] OR "fruit"[tiab] OR "vegetables"[MeSH Terms] OR "vegetables"[tiab])<br><br>AND (rural OR underserved OR "under served" OR under-served OR non-urban OR "non urban" OR underdeveloped OR under-developed OR "under developed" OR "low socioeconomic status" OR "Socioeconomic Factors")<br><br>AND (preschool[Title/Abstract] OR pre-school[Title/Abstract] OR "pre school"[Title/Abstract] OR "child, preschool"[MeSH Terms] OR toddler[Title/Abstract] OR "child development center"[Title/Abstract] OR "early childhood"[Title/Abstract])	AND (rural OR underserved OR "under served" OR under-served OR non-urban OR "non urban" OR underdeveloped OR under-developed OR "under developed" OR "low socioeconomic status" OR "Socioeconomic Factors" OR disadvantaged)<br><br>AND (preschool OR pre-school OR "pre school" OR OR toddler OR "child development centers" OR "early childhood" OR "preschool children")		
**Date of search**	2 April 2019	2 April 2019	2 April 2019	5 February 2019
**# records retrieved before de-dup.**	12381	402	1468	2
